# Influence of Diet and Growth Conditions on the Carbon and Nitrogen Stable Isotopic Composition of *Aspergillus niger* Mycelium: Insights for Fungal Chitosan Characterization

**DOI:** 10.3390/molecules30204142

**Published:** 2025-10-21

**Authors:** Matteo Perini, Raffaele Guzzon, Silvia Pianezze, Francesca Violardo, Roberto Larcher

**Affiliations:** Fondazione Edmund Mach, Via E. Mach n. 2, 38098 San Michele all’Adige, TN, Italy; raffaele.guzzon@fmach.it (R.G.); violardo.francesca@gmail.com (F.V.); roberto.larcher@fmach.it (R.L.)

**Keywords:** mycelium, stable isotope, *Aspergillus niger*, diet, stable isotope fractionation

## Abstract

This study investigates, for the first time, the relationship between carbon (*δ*^13^C) and nitrogen stable isotopic composition of *Aspergillus niger* mycelium, used as chitin and chitosan sources, and the fungus diet under controlled cultivation conditions. Four diets were tested, combining different carbon (C3- and C4-glucose) and nitrogen sources (KNO_3_ and NH_4_Cl). Results showed that carbon sources significantly influenced *δ*^13^C values of the mycelium: C4-glucose diets led to more negative *Δ*^13^C values (*δ*^13^C_MYCELIUM_-*δ*^13^C_DIET_) compared to C3-glucose diets. Nitrogen sources also affected isotopic fractionation, with KNO_3_ leading to negative *Δ*^15^N (*δ*^15^N_MYCELIUM_-*δ*^15^N_DIET_) and NH_4_Cl yielding positive *Δ*^15^N. Conversely, pH and temperature showed negligible effects on *δ*^15^N, while continuous aeration during growth significantly decreased *δ*^15^N, possibly due to partial assimilation of atmospheric nitrogen. These findings demonstrate that both nutrient and cultivation parameters can modulate the isotopic fractionation in *A. niger*, particularly for nitrogen. Although a direct correlation between diet composition and *δ*^15^N could not be established, this work provides the first experimental link between fungal metabolism and its isotopic fingerprint. The results offer a scientific foundation for applying stable isotope ratio analysis to authenticate and trace fungal-derived chitin and chitosan, with potential applications in food and winemaking industries.

## 1. Introduction

Chitosan is a natural biopolymer that, thanks to its chemical and physical properties, has recently found various applications in technological and industrial fields. In recent years, its use has also been proposed in winemaking, mainly as an antimicrobial, antioxidant, clarifying and chelating agent [1]. Commercially, this biopolymer is cost effectively obtained through the chemical deacetylation of chitin, which is the structural element of animal exoskeletons and fungal cell walls [2]. To date, the major source of industrial chitin comes from marine food production wastes, mainly crustacean shells, e.g., shrimp, crab or krill shells [3]. However, the International Organisation of Vine and Wine (OIV), through resolution OIV-Oeno 368-2009 [4], exclusively authorizes chitosan of fungal origin for winemaking purposes, which is normally obtained from citric acid production waste [5], while the animal-derived one is forbidden. The reason for this decision lies in the fact that the crustaceans can cause allergic reactions such as anaphylaxis [6], due to proteins like tropomyosin [7].

To verify the fungal origin of chitosan and prevent fraudulent substitution with animal-derived material, the OIV has outlined various analytical methods. Chitosan of fungal origin may simultaneously meet specific thresholds: residual glucan content > 2%, settled density ≥ 0.7 g/cm^3^, and viscosity < 15 cP in a 1% acetic acid solution. However, these parameters are not always concurrently satisfied and can potentially be manipulated to meet the specifications. Furthermore, these methods are labour-intensive, require over three hours per sample, and rely on instruments not always available in standard laboratories. Consequently, there is a growing demand for faster, more automated, and reliable approaches for chitosan authentication.

In response to these limitations, Perini et al. introduced a promising alternative based on stable isotope ratio (SIR) analysis of elements such as carbon and nitrogen, which enables the distinction between fungal- and animal-derived chitosan [8].

The method has been included in 2025 in the chitosan monograph (Resolution OIV-Oeno 368-2009) among the analytical techniques that can be used to guarantee its fungal origin with the OIV/OENO-SPECIF 23-728 resolution. More recently, Claverie et al. helped to define the isotopic ranges that characterize the two chitosan origins [9]. The author speculated that the isotopic differences they found could be due to the different sources of C, N, O and H used by either the fungus or the crustaceans, which are characterized by different isotopic compositions. For instance, the carbon absorbed by the fungus strain during its growth derives almost exclusively from either beet or cane sugar and could therefore likely reflect the typical carbon isotopic ratio (*δ*^13^C) of a C3 (from −29‰ to −25‰) or a C4 (between −14‰ and −12‰) plant [10]. As reported by Claverie et al., the *δ*^13^C of C3-fed fungal chitosan varied from −25.6‰ to −24.8‰, while the same parameter varied from −14.2‰ to −12.9‰ for C4-fed fungal chitosan [9]. Moreover, if the value of *δ*^13^C falls between −25.1‰ and −24.9‰, it would be necessary to proceed with the evaluation of the *δ*^15^N parameter, which must be above +2.7‰ to classify chitosan as from fungi. On the other hand, an average *δ*^13^C value of −21.1 ± 2.0‰ was reported for chitosan derived from crustaceans, which feed on marine phytoplankton having a typical *δ*^13^C of −20‰.

Both studies of Perini et al. [8] and Claverie et al. [9] were conducted exclusively on finished products (animal-derived chitosan from shrimp, crab and squid and fungal-derived chitosan from *Aspergillus niger* and *Agaricus bisporus*) provided by different producers. The isotopic relationship among the living organism (e.g., the fungus *A. niger*) fed on a specific diet, the diet composition and the chitin/chitosan produced by the organism itself has never been studied in detail, but it has only been inferred based on studies on terrestrial and aquatic organisms, such the one reported by DeNiro et al. [11]. Henn et al. [12] and Ruess et al. [13] in their respective studies on fungi (*δ*^13^C and *δ*^15^N variability), highlighted that the isotopic fractionation between mycelia and diet could vary depending on the chemoheterotrophic organism under study.

In this study, we focused on the initial stage of chitosan production, providing experimental evidence that links the isotopic composition of the *A. niger* diet to the stable isotopic signature (*δ*^13^C and *δ*^15^N) of the resulting fungal mycelium—the primary source of chitin. The effects on the subsequent stages, such as chitin extraction, purification, and deacetylation, were not considered here.

We investigated how different carbon and nitrogen sources, together with cultivation parameters such as temperature, pH and aeration, can influence the isotopic fractionation during fungal growth. This allowed us to identify the conditions that most strongly affect the isotopic composition of the final fungal biomass.

Specifically, the study aimed to quantify the extent of the isotopic fractionation under varying nutritional and environmental conditions and to determine which parameters have the greatest impact on *δ*^13^C and *δ*^15^N values. By establishing this relationship between the isotopic composition of the growth substrates and the resulting fungal signature, our work provides the first experimental evidence supporting the use of stable isotope ratios to authenticate the origin of chitosan.

## 2. Results and Discussion

### 2.1. Isotopic Analysis of Starting Spore Strain

To evaluate the possible correlation between the isotopic composition of the mycelium produced by *A. niger* and the starting spore of the fungus itself (see Section 3.1.2), the latter was isotopically analysed. Three replicates of the same starting spore sample were considered. The results of the *δ*^13^C and *δ*^15^N analysis are shown in Table 1.

The isotopic composition of the starting spore (average *δ*^13^C = −13.2‰ and *δ*^15^N = −1.3‰) doesn’t seem to be the major factor contributing to the *δ*^13^C and *δ*^15^N of the fungus mycelium.

In the case of diet A, the fungus was provided with a Glucose 1 (*δ*^13^C = −10.9‰) which resulted in a final mycelium having more negative *δ*^13^C (mean *δ*^13^C = −17.7‰, Table S1). In case the starting spore represented a relevant contribution to the *δ*^13^C of the final mycelium, a much less negative value would be expected. It can be therefore stated that the isotopic contribution of the starting spore is completely negligible and that the isotopic value of the final mycelium is solely correlated with the sources supplied to the fungus during its growth.

### 2.2. Estimation of the Inter-Days Repeatability

The replicates of both A and B diet sampled at different times (Table S1) were used to estimate the inter-days repeatability of the whole method (including the inoculum preparation, the mycelium growing and separation and finally in the stable isotope analysis). At 95% confidence level, the standard deviation of repeatability (s_r_) was estimated to be 0.3‰ for *δ*^13^C and 0.6‰ for *δ*^15^N. Correspondingly, the repeatability (r), calculated as 2.8 × s_r_, was 0.8‰ for *δ*^13^C and 1.8‰ for *δ*^15^N. Deviations among the isotopic values below these limits can therefore be considered as not significant.

### 2.3. Experiment 1 Test 1: Effect of Different Diets

**Figure 1:**
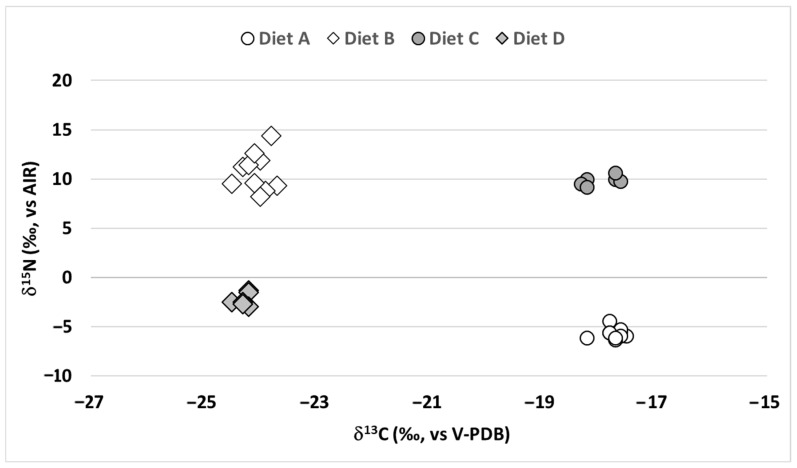
Plot of *δ*^13^C vs. *δ*^15^N of the grown mycelium (chitin source) samples obtained from the fungus *Aspergillus niger* fed with four different diets.

The results of the carbon and nitrogen isotopic analyses of the mycelium samples obtained by growing of *A. niger* with four different diets (Diet A–D) are presented in Figure 1.

#### 2.3.1. Carbon Isotopic Ratio (δ^13^C) Shift Between the Diet and the Mycelium Produced by the *Aspergillus niger* Fungus

The mycelium obtained from *A. niger* fed on diets A and C (Figure 1 and Supplementary Table S1), both including a C4-carbon source (Glucose 1, *δ*^13^C = −10.9‰), exhibited a lower (more negative) *δ*^13^C than the diet. The *Δ*^13^C, defined as (*δ*^13^C_MYCELIUM_-*δ*^13^C_DIET_), was calculated as −6.8‰ and −7.1‰ for diets A and C, respectively. Conversely, mycelium from *A. niger* fed on diets B and D (Figure 1), both including a C3-carbon source (Glucose 2, *δ*^13^C = −23.7‰), showed smaller differences between the *δ*^13^C of diet and mycelium. The *Δ*^13^C values for diets B and D resulted in −0.4‰ and −0.6‰, respectively.

Earlier studies in fungi provided evidence of specifical biochemical pathways resulting in strong fractionation effects [14]. The enrichment in either the heavy or the light carbon isotope in fungal biomass compared to the bulk substrate (which is comparable to the just mentioned parameter *Δ*^13^C) of up to 7‰ was previously reported by Will et al. [15]. Moreover, deviations comparable to those found in this study were previously reported in other fungal species by Henn et al. [12]. For instance, when exposed to the same C3-based diet, the fungus *Cryptoporus volvatus* showed negligible enrichment with respect to its diet (+0.67‰), whereas *Marasmius androsaceus* and *Suillus granulatus* exhibited significant isotopic enrichments, having *Δ*^13^C (described in the study as isotopic discrimination and calculated as *δ*^13^C_FUNGAL BIOMASS_-*δ*^13^C_BLANK MEDIUM_) values of +4.87‰ and +4.91‰, respectively. On the other hand, in the same work and in disagreement with the results shown in this study, Henn et al., reported that the isotopic enrichment became negligible when the fungi were subjected to a C4-based diet [12].

According to Rossmann et al., glucose showed a pronounced isotopic asymmetry, which is more evident in sugars derived from C3 than from C4 plants [16]. The *Δδ*^13^C values, defined by the authors as deviations relative to the measured average value for the glucose molecule, were calculated and used to compare the average *δ*^13^C of the glucose molecule to the *δ*^13^C of every carbon atom in the structure (analysed after chemical degradation).

**Figure 2:**
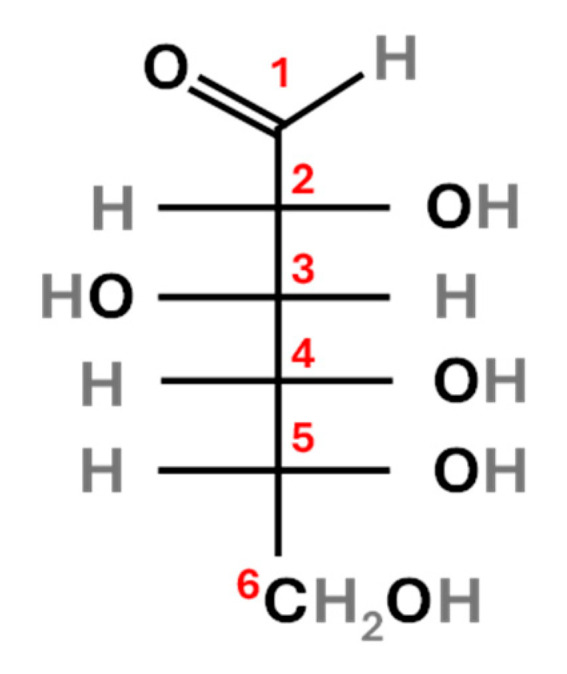
Glucose structure: carbon atoms are labelled in red (1–6).

The calculation of the *Δδ*^13^C values revealed that for carbon 3 (Figure 2), C3 glucose was ^13^C-enriched compared to molecular *δ*^13^C, with a *Δδ*^13^C = 2.2‰, while C4 glucose had a *Δδ*^13^C = 1.2‰. For carbon 6 (Figure 2), C3 glucose was ^13^C-depleted compared to molecular *δ*^13^C, with a *Δδ*^13^C = −5.2‰, whereas C4 glucose had *Δδ*^13^C = −4.0‰. This asymmetry can explain the isotopic deviations observed in fungi fed on C3 vs. C4 diets, as suggested by Henn et al. [12]. In particular, the authors stated that carbon fractionation was related to the selective uptake of ^13^C-enriched C rather than from the selective loss of ^12^C from fungal biomass through depleted CO_2_. Once they stated that the focus to understand C fractionation was to be put on its uptake, they proposed a model called dual-uptake hypothesis. Briefly, they suggest two alternative routes in fungi C uptake, resulting in differential isotopic discrimination. In the first one, the hexose molecules are brought into the cell without catabolism, resulting in no C fractionation; in the second one, the hexose molecules are broken down extracellularly into triose fragments though two separate routes characterised by different rates, therefore resulting in C fractionation. Despite the requirement for confirmation of this hypothesis, it would support the results obtained in the present study. Moreover, in a following study of Henn, [17] the same authors assessed that the exact means by which ^13^C is selectively taken into the fungal cell from the medium is unclear. Therefore, to fully understand the metabolic pathway that the sugars undergo from source to fungus further studies are required.

Henn et al. also highlighted significant isotopic effects associated with sucrose utilization by different species of basidiomycetes [12]. These effects are species-specific and vary consistently depending on whether the sucrose originates from a C3 or C4 carbon source. As such, it is crucial to study the isotopic behaviour of individual chemoheterotrophic organisms independently.

The shift toward more negative *δ*^13^C values during mycelium biosynthesis (from −10.9‰ to −17.7‰ in the C4 diet, and from −23.7‰ to −24.1‰ in the C3 one) was consistent with previously reported fractionation processes occurring during different biosynthetic pathways [18]. Furthermore, certain types of bacteria use carbon sources with specific *δ*^13^C signatures or possess metabolic pathways that yield specific *δ*^13^C values, which can support their identification [18].

The results demonstrate that under identical growth conditions (temperature and pH) the *δ*^13^C signatures of *A. niger* mycelium (chitin source) can be directly traced back to the isotopic composition of the diet with deviations depending on the type of carbon source (C3- or C4- derived glucose).

#### 2.3.2. Nitrogen Isotopic Ratio (*δ*^15^N) Shift Between the Diet and the Final Mycelium Produced by the *Aspergillus niger* Fungus

As for *δ*^15^N, the mycelium obtained from *A. niger* fed on diets A and D (Figure 1), both including potassium nitrate as nitrogen source, showed lower *δ*^15^N values compared to the diet. The *Δ*^15^N, defined as (*δ*^15^N_MYCELIUM_-*δ*^15^N_DIET_), was −7.0‰ and −3.6‰ for diets A and D, respectively. In contrast, for diets B and C (Figure 1), both including ammonium chloride as nitrogen source, an unexpected positive difference between the *δ*^15^N of the mycelium and the diet was observed. The *Δ*^15^N values resulted +12.5‰ for diet B and +11.7‰ for diet C.

The enrichment in either the heavy or the light nitrogen isotope in fungal biomass compared to the bulk substrate (which is comparable to the just mentioned parameter *Δ*^15^N) of up to 4‰ was previously reported by Will et al. [15] and forest basidiomycetes were also found to become either enriched or depleted in ^15^N relative to atmospheric nitrogen.

A trend similar to that found in this study was reported by Emmerton et al. in a study on nitrogen assimilation and isotopic fractionation in the mycorrhizal fungi *Paxillus involutus* and *Leccinum scabrum* [19]. When supplied with ammonium sulphate, the *δ*^15^N of the nitrogen (N_2_) assimilated by *P. involutus* and *L. scabrum* displayed *Δ*^15^N values of +4‰ and +3‰, respectively. On the other hand, when provided with calcium nitrate the *δ*^15^N of the N_2_ assimilated by both fungi was significantly lower than the dietary one. In the same study, a different nitrogen isotopic fractionation was observed for an ericoid mycorrhizal fungus (ERM) [19]. When the ERM fungus was grown with calcium nitrate, no fractionation relative to the nitrogen source was observed, whereas a *Δ*^15^N of −15‰ was reported when including ammonium sulphate in the diet. Although these results are difficult to fully explain, Emmerton et al. [19] suggest that nitrogen fractionation during assimilation largely depends on the pathways preferentially used by the fungal species considered.

An analogous trend in *δ*^15^N values was also reported by Högberg et al. in their study on nitrogen isotopic fractionation in fungal species forming symbiotic associations (mycorrhizae) with Scots pine (*Pinus sylvestris*) [20]. Högberg et al. examined variations in *δ*^15^N values among three fungal species during nutrient transport from soil to plant, using potassium nitrate (*δ*^15^N = 0.6 ± 0.0‰) and ammonium chloride (*δ*^15^N = −0.5 ± 0.0‰) as nitrogen sources in solutions with pH ≈ 7.4 and 4, respectively. The authors reported *δ*^15^N values in some cases more negative and in others more positive than the nitrogen source, regardless of the diet. For example, *Suillus bovinus* exhibited more positive *δ*^15^N values than the nitrogen source with an ammonium chloride-bases diet, while with a potassium nitrate-based diet, *δ*^15^N values were more negative. This pattern supports the *δ*^15^N trends observed in the present study for *A. niger* grown with different nitrogen sources. The authors argued that the larger isotopic effect (which is related, in the present study, to the higher *Δ*^15^N) observed for the diets including the ammonium chloride rather than the potassium nitrate is to be expected, because of the larger relative difference in mass if ^15^N replaces ^14^N in the ammonium chloride rather than in the potassium nitrate.

It is worth making an additional observation on the difference in the *Δ*^15^N of diets including either the ammonium or the nitrate N source. The higher *Δ*^15^N in diets B and C (ammonium) than A and D (nitrate) could be related to the efficiency of the N source. A study on the removal of multiple nitrogenous wastes by the fungus *A. niger* showed that ammonium was metabolised rapidly prior to nitrite, since the latter was a less efficient source of nitrogen for cell growth compared to the former. It must be noticed that nitrite is not nitrate, therefore even though an exact comparison cannot be made, parallelism can still be noticed. The higher N uptake could be therefore related to a more consistent fractionation, as already stated in plants [20]. In support to this affirmation, Högberg et al. found out that plants given nitrate took up 12–33% of the N supplied and had less fractionation with respect to the source (0.0‰–1.7‰), whereas plants given ammonium took up 18–86% of the N supplied and had higher fractionation with respect to the source (0.9‰–5.8‰). Ruess et al. reported similar *δ*^15^N trends in fungi such as *Laccaria laccata*, *Agrocybe gibberosa*, and *Chaetomium globosum* [13]. They observed both positive (e.g., *C. globosum Δ*^15^N = 0.3‰, *A. gibberosa Δ*^15^N = 0.8‰) and negative (e.g., *L. laccata Δ*^15^N = −2.1‰) deviations in *δ*^15^N values between the fungus and the diet (e.g., potato dextrose or Pachlewska agar). Possible sources of variation in *δ*^15^N fractionation were attributed to differences in the type and quality of food, as well as to the metabolism and physiological state of the fungal species [13].

As Brauer et al. noted, factors such as pH, temperature, light exposure, and the nitrogen source included in the diet can influence mycelium biosynthesis in *Aspergillus* fungi [21]. In response to such stressors, the fungal cell wall, composed of mycelium, undergoes continuous remodelling to tolerate adverse extracellular conditions, ensuring growth and reproduction while avoiding cell death [22]. Furthermore, condition-specific alterations in the *Aspergillus* cell wall may occur, potentially leading to increased mycelium production and/or redistribution within the organism [23].

The results indicate that the nitrogen source in the substrate significantly influences both the direction and magnitude of nitrogen isotopic fractionation in the mycelium produced by the *A. niger*. However, the observed differences among treatments cannot be attributed solely to the chemical nature of the nitrogen source (nitrate or ammonium). Other factors, such as nitrogen assimilation efficiency, growth conditions, and internal metabolic processes, are likely to contribute to the observed isotopic variability.

Therefore, based on the available data, a direct and generalizable relationship between the diet and the *δ*^15^N values of chitin cannot be established. Instead, the nitrogen isotopic fractionation in *A. niger* appears to reflect the complex interaction between the type of nitrogen source and the fungus specific physiological responses to environmental and nutritional conditions.

### 2.4. Temporal and Condition-Dependent Fractionation of Carbon and Nitrogen Isotopes in Aspergillus niger Mycelium

Despite the hypotheses presented in Section 2.3.1 and Section 2.3.2, which tried to explain the pathways on which nitrogen and carbon fractionation in fungi are based, many authors agreed in affirming that the mechanism of C and N fractionation between the fungus and its source is still far from being understood (Henn & Chapela, [14], Henn et al., [17]). Therefore, to better understand the mechanisms underlying the variations in *δ*^13^C and *δ*^15^N between the diet and the mycelium produced by *A. niger*, fractionation tests described in Section 2.4.1 were designed and conducted. Since diets A and D (including potassium nitrate) were cultured in a pH ≈ 7 broth, while diets B and C (including ammonium chloride) were cultured in a pH ≈ 5 broth, an additional experiment (Section 2.4.2) was conducted to evaluate whether pH effects could explain the observed *δ*^15^N differences.

#### 2.4.1. Experiment 1 Test 2: Monitoring over the Time

To better understand the overtime changes in the *δ*^13^C and *δ*^15^N values of mycelium during its production by the fungus *A. niger*, fractionation tests were designed and conducted using diets A and B as reported in Section 3.1.4. The results of the duplicate experiments on diets A and B are reported in Table 2 (missing data correspond to a missed mycelium sampling).

Considering the estimated repeatability (see Section 2.2), the results for the *δ*^13^C showed negligible variation (*p* > 0.05) over the collections, both for diet A and B. The *δ*^13^C of the mycelium produced by the fungi remained steady from the first sampling, which occurred 2–4 days after inoculation, to the last one.

A different trend was found for *δ*^15^N. Unlike *δ*^13^C, the *δ*^15^N values progressively increased for diet B, while decreased for diet A. The variation in the *δ*^15^N values indicates nitrogen fractionation processes during mycelium biosynthesis. As reported in paragraph 2.3.2, the variation in *δ*^15^N can be caused by the different conditions in which the *A. niger* fungus grows, particularly the nitrogen source administered in the diet (potassium nitrate vs. ammonium chloride).

#### 2.4.2. Experiment 2: Effect of pH, Temperature and Airflow on the *δ*^15^N Values

To understand the possible effects induced by some fermentation parameters on the *δ*^15^N, two different experiments were set up. In the first one, the effect of temperature and pH were studied. The fungi fed on diet B and kept at 25 °C produced a mycelium having mean *δ*^15^N values of +10.6‰, +9.5‰ and +9.5‰ at pH 5.3, 7 and 8, respectively. The *δ*^15^N measured at different pH values were not statistically different, considering the repeatability estimated in Section 2.2.

On the other hand, temperature had a significant impact on the *δ*^15^N mycelium produced by *A. niger* fed on diet B and growing in a pH = 5.3 broth. The *δ*^15^N of mycelium produced at 15 °C (+6.4‰) was statistically different to the values measured at 25 °C (+13.6‰) and 40 °C (+14.5‰) (*p* < 0.05). The lower temperature of 15 °C contributed to slowing down the growth process and led to as high isotopic values as the other temperatures (Table 3).

Finally, the effect of constant airflow applied during the fungi growth was considered. The pH was monitored and remained steady all the test long (pH = 5.3). For both diets, a statistical difference (*p* < 0.01 and Tukey test) was highlighted between the *δ*^15^N of mycelium samples produced under or without the airflow. Regardless of the diet, higher *δ*^15^N values were found for mycelium produced without the use of airflow (Table 3). This may be because, as hypothesized by some authors, *Aspergillus* species can fix the atmospheric nitrogen.

Pannington et al. reported that the ability of fungi to absorb atmospheric nitrogen has been an open question for years [24]. Lipman’s experiments seemed to demonstrate this ability for the *Aspergillus* species [25], but subsequent studies often led to opposite conclusions [26]. The atmospheric nitrogen *δ*^15^N is equal to 0‰ (standard AIR). Its assimilation by the fungus could justify a decrease in the *δ*^15^N value recorded in samples subjected to airflow, which improves atmospheric nitrogen dispersion in the broth. Further studies are required to better understand this phenomenon, eventually considering additional nitrogen and carbon sources, as well as fungal species other than *Aspergillus*.

## 3. Materials and Methods

### 3.1. Description of Samples and Processes

#### 3.1.1. Ingredients

The *A. niger ATCC6275* was purchased from the American Type Culture Collection (Manassas, VA, USA) in dried form, rehydrated according to the supplier’s instructions and allowed to grow in Potato Dextrose Broth (PDB, Oxoid, Basingstoke, UK) for 48 h at 25 °C. Then, the culture was supplemented with 20% *v*/*v* glycerol (Sigma Aldrich, Saint Louis, MO, USA) and stored at −80 °C until the tests. Water employed in the tests was twice demineralized by a Water Purifier (Stedim Arium 611DI, Sartorius AG, Göttingen, Germany) and sterilized at 121 °C for 15 min in VE-95 autoclave (Systec GmbH & Co. KG, Linden, Germany). Potassium nitrate (KNO_3_, *δ*^15^N = 1.3‰) and ammonium chloride (NH_4_Cl, *δ*^15^N = −1.8‰) (Merck, Darmstadt, Germany) were used as nitrogen sources, while two glucose samples (CARLO ERBA Reagents S.r.l., Milan, Italy) with different *δ*^13^C values were used as carbon sources (Glucose 1: *δ*^13^C = −10.9‰; Glucose 2: *δ*^13^C = −23.7‰). The two samples were chosen to mimic a C4 and C3 diet, respectively. The isotopic values were obtained by analysing the different ingredients in triplicate.

#### 3.1.2. Preparation of the Starting Spore

The fungus culture (1 mL having a nominal concentration of 10^7^ CFU/mL) was reactivated in 200 mL of PDB for 24 h at 25 °C, then multiplied for a variable interval between 3 and 30 days in growing volume and in the same conditions.

To analyse the isotopic composition of *A. niger* and evaluate their potential influence on subsequent mycelium analyses, 10 Petri dishes (9 cm diameter) were filled with potato dextrose agar (PDA, Oxoid, Basingstoke, UK) growth medium, onto which 1 mL of spore was spread. Dishes were incubated for 7 days at 25 °C, resulting in a complete covering of the surface by mould mycelium. After incubation, the mycelium was removed by a sterile stainless-steel spatula and divided into 3 Eppendorf tubes. The mycelium was then washed with deionized water and centrifuged (4500 rpm for 5 min with Thermo centrifuge KR4, Bremen, Germany) three times. The resulting samples were subsequently frozen and lyophilized.

#### 3.1.3. Experiment 1 Test 1: Effect of Different Diets

The production of citric acid from *A. niger* results in approximately 80,000 tons/year of mycelial waste materials [27]. These fungal wastes represent a free natural source of chitin and chitosan. For this reason, in this study, the laboratory-synthesized mycelium samples were obtained from *A. niger*, which is considered as the commercially most used fungi for chitosan production. To evaluate the correlation between the isotopic composition of the diet provided to the growing fungus and of the mycelium used as chitin source finally produced by the fungus itself, four specific diets characterized by different sources of nitrogen and carbon were formulated.

For this purpose, 1 g of *A. niger* fungal mycelium was inoculated on the culture media (1 L for each test) consisting of water and specific carbon and nitrogen sources, in concentrations which aimed to simulate the diet provided to the fungus in common industrial processes. The complete description of the four diets (Diet A–D) is reported in Table 4.

After inoculation, the different media were incubated at 25 °C, and the fungal mycelium produced 10 days after inoculation was sampled.

For diets A and B, 5 samples of *A. niger* were considered for each one. The experiment was subsequently replicated, resulting in a total of 10 replicates for diets A (Table S1, samples 1–10) and B (Table S1, samples 11–20). For diets C and D, 3 samples of *A.niger* were considered for each diet. The experiment was duplicated, resulting in a total of 6 replicates for diets C (Table S1, samples 21–26) and D (Table S1, samples 27–32).

#### 3.1.4. Experiment 1 Test 2: Monitoring over the Time

Diets A and B were finally selected to monitor the *δ*^15^N and *δ*^13^C variation in the mycelium produced by the *A. niger* fungus over time. For this purpose, the fungus inoculated in the culture medium was allowed to grow for 5–12 days, and the produced grown mycelium was subsequently sampled 4 times at two-day intervals. Since a small part of the fungal product needed to be sampled multiple times, it was necessary to use a larger amount of starting material (both *A. niger* and culture medium). Therefore, it was decided to simulate the industrial conditions applied to the bioreactors used for chitosan production, using a 3 L volume of culture medium inoculated with 5 mL of *A. niger* culture subject to a continuous air stream at 25 °C. In this part, two fungus samples were considered for each diet, repeating the experiment twice.

#### 3.1.5. Experiment 2: Effect of the Variation in pH, Temperature and Presence or Absence of a Constant Airflow on *δ*^15^N Value

The effect of temperature and pH variation were evaluated separately in two different experiments. The fungi *A. niger* were given diet B and grown for 10 days. To evaluate the pH effect, the fungi were grown at a constant temperature of 25 °C in three different culture broths adjusted to pH 5.3, 7.0 and 8.0. Three replicates for each pH value were considered (see Table 3, Test 1). To evaluate the temperature effect, the fungi were grown for 10 days at a constant pH of 5.3 in three culture broths at constant temperatures of 15 °C, 25 °C and 40 °C. Three replicates for each temperature value were considered (see Table 3, Test 2). Therefore, the effect of the air stream applied during the growth was evaluated. The fungi were fed with diets B and C, both subject and not subject to a continuous air flow (1 mL/min). Three replicates for each combination (diet B–no flow; diet B–air flow; diet C–no flow; diet C–air flow) were considered (see Table 3, Test 3).

#### 3.1.6. Mycelium Sample Isolation

The fungal mycelia produced by *A. niger* were washed with water using an ULTRA TURRAX^®^ IKA^®^ T25 digital and centrifuged three times (Thermo centrifuge at 4100 rpm for 5 min). The resulting mycelium samples were then freeze-dried with an ALPHA I-5 ChrisA 5Pascal lyophilized (Duration 24 h). Finally, they were homogenized using a mortar and pestle in liquid nitrogen.

### 3.2. Stable Isotope Ratio Analysis

For the determination of *δ*^15^N and *δ*^13^C values, samples were weighed 0.8 mg in tin capsules (4 mm diameter, 6 mm height) using a microbalance (XM1000P Sartorius Lab Instruments GmbH & Co. KG, Göttingen, Germany). Each capsule was closed with forceps and pressed to eliminate residual air. To simultaneously perform *δ*^15^N and *δ*^13^C analyses, tin capsules were inserted into an Elemental Analyzer (EA) (NC analyser FLASH EA 1112 SERIES, Thermo Scientific, Waltham, MA, USA) for total sample combustion. The gasses (CO_2_ and N_2_) resulting from the combustion were then transferred through a helium stream to an Isotope Ratio Mass Spectrometer (IRMS) (Finnigan DELTA XP, Thermo Scientific) for the isotopic ratio measurement. The *δ*^15^N and *δ*^13^C values were calculated using two internal reference standards (casein ST1: *δ*^15^N = 6.56‰ and *δ*^13^C = −23.51‰; casein ST2: *δ*^15^N = 7.38‰ and *δ*^13^C = −21.98‰), calibrated against international standard reference materials. For ^13^C/^12^C analysis, the reference standards used were fuel NBS-22 (*δ*^13^C = −30.03‰), sucrose IAEA-CH6 (*δ*^13^C = −10.45‰) (IAEA—International Atomic Energy Agency, Vienna, Austria), and L-glutamic acid USGS 40 (*δ*^13^C = −26.39‰ and *δ*^15^N = −4.52‰) (U.S. Geological Survey, Reston, VA, USA); for ^15^N/^14^N analysis, the standards used were glutamic acid USGS 40 (*δ*^13^C = −26.39‰ and *δ*^15^N = −4.52‰) (U.S. Geological Survey, Reston, VA, USA) and potassium nitrate IAEA-NO3 (*δ*^15^N = 4.7‰) (IAEA—International Atomic Energy Agency, Vienna, Austria).

The instrumental precision, expressed as reproducibility limit calculated as 2 * rad2 * SD_reproducibility_ of the same sample, was 0.3 for both *δ*^15^N and *δ*^13^C.

The measured isotope ratios are reported in the delta (*δ*) notation corresponding to the relative deviations of the molar ratio (*R*) of the heavy elements (*^i^E* i.e., ^13^C) to light elements (*^j^E* i.e., ^12^C) isotopes in the samples from those in international standards V−PDB (Vienna−Pee Dee Belemnite) for *δ*^13^C and Air (atmospheric N_2_) for *δ*^15^N, as shown in the following equation:

(1) $${\delta_{ref}(}^{i}E{/}^{j}E,sample)=\left[\frac{R{(}^{i}E{/}^{j}E,sample)}{R{(}^{i}E{/}^{j}E,ref)}\right]-1$$

where, *ref* is the international measurement standard, *sample* is the analysed sample, and *^i^E*/*^j^E* is the ratio of heavier to lighter isotopes.

The delta values are here multiplied by 1000 and expressed in the more common unit "per mil" (‰) rather than, as required by the International System of Units (SI), in milliurey units (mUr).

### 3.3. Statistics

The statistical analysis of the isotopic results was performed using the Statistica program version 14.0.1.25. Data normality was tested using the Kolmogorov–Smirnov and Lilliefors tests. To evaluate significant differences between groups, a one-way ANOVA was performed, with Tukey’s test as a post hoc test. The threshold *p*-value for significant differences was set at 0.05. Pearson’s correlation coefficient was used for assessing the linear correlation between two parameters.

## 4. Conclusions

This study investigated for the first time the correlation between the isotopic values of *A. niger* fungal mycelium used as a chitin source, a precursor of chitosan, and those of the diet supplied to the fungus for its growth.

Different combinations of carbon (glucose from C3 and C4 plants) and nitrogen sources (potassium nitrate and ammonium chloride) were considered. The use of glucose from C4 plants led to larger absolute *Δ*^13^C values (defined as *δ*^13^C_MYCELIUM_-*δ*^13^C_DIET_) than glucose from C3 plants and repeated experiments showed that this deviation remained steady over time.

The nitrogen source affected the *δ*^15^N values of the mycelium differently, with positive *Δ*^15^N (defined as *δ*^15^N_MYCELIUM_-*δ*^15^N_DIET_) values measured when the ammonium chloride was considered and negative *Δ*^15^N values when potassium nitrate was taken as the primary nitrogen source. *δ*^15^N did not seem to be affected by the broth pH or the temperature usually applied in industrial procedures (higher than 25 °C), but it was clearly affected by the application of an airflow during the growth.

Although the fractionation effects on the subsequent stages of chitosan production (i.e., chitin extraction, purification, and deacetylation to chitosan) have not been evaluated yet, this study represents the first step toward understanding how the isotopic composition of *A. niger* mycelium relates to its growth conditions and nutrient sources. Carbon isotopic ratios (*δ*^13^C) clearly reflected the isotopic composition of the carbon source, confirming the reliability of *δ*^13^C as an indicator of dietary carbon origin. Conversely, nitrogen isotopic ratios (*δ*^15^N) showed more complex fractionation behaviour: while the nitrogen source influenced the direction of isotopic enrichment (negative *Δ*^15^N for nitrate and positive *Δ*^15^N for ammonium), no simple or direct correlation could be established between the diet composition and the *δ*^15^N of the fungal mycelium.

These findings suggest that nitrogen isotopic fractionation in *A. niger* does not only depend on the chemical form of nitrogen, but also on the fungus metabolism and physiological responses to cultivation conditions such as aeration. Overall, this work provides the first experimental framework for interpreting isotopic variability in fungal chitin and its derived chitosan, offering a scientific basis for the development of isotopic tools for authentication and traceability of fungal-derived biopolymers. Although exploratory in nature, the study delivers fundamental insights that can guide future research aimed at expanding the dataset and strengthening the statistical robustness of isotopic models for chitosan origin determination.

## Figures and Tables

**Table 1:** Carbon (*δ*^13^C) and nitrogen (*δ*^15^N) values for three replicates of the starting spore sample.

	*δ*^13^C (‰, vs. V-PDB)	*δ*^15^N (‰, vs. Air)
1	−12.4	−1.2
2	−13.2	−1.4
3	−14.1	−1.3
Mean	−13.2	−1.3
St. Dev.	0.9	0.1

**Table 2:** Results of the isotopic fractionation tests for carbon (*δ*^13^C) and nitrogen (*δ*^15^N) of the mycelium produced by the fungus *Aspergillus niger* fed with diets A and B. For each test, mycelium (chitin source) was sampled 4 times every other day (Sampling No. 1–4) from two fungi samples (Sample 1 and 2).

Diet A	Sample	Sampling No. 1	Sampling No. 2	Sampling No. 3	Sampling No. 4
*δ*^15^N (‰, vs. Air)	1	-	−1.2	−2.3	−5.6
	2	0.6	−1.6	−2.4	−4.6
*δ*^13^C (‰, vs. V-PDB)	1	-	−17.5	−17.6	−17.5
	2	−17.9	−17.9	−17.6	−18.2
Diet B	Sample	Sampling No. 1	Sampling No. 2	Sampling No. 3	Sampling No. 4
*δ*^15^N (‰, vs. Air)	1	2.3	7.3	7.4	10.5
	2	5.7	8.9	9.3	10.8
*δ*^13^C (‰, vs. V-PDB)	1	−24.0	−24.4	−24.4	−24.4
	2	−24.2	−24.4	−24.4	−24.4

**Table 3:** Effect of temperature, pH and airflow on mycelium *δ*^15^N value (mean of three replicates each); diet B was considered to study the effect of temperature and pH, while diets B and C were considered to study the effect of the airflow; different letters indicate statistically different results (*p* < 0.05).

Test	Diet	Temperature (°C)	pH	Airflow	Mean *δ*^15^N (‰, vs. Air)		St. Dev.
1	B	25	5.3	no	10.6	a	0.6
	B	25	7.0	no	9.5	a	0.8
	B	25	8.0	no	9.5	a	0.7
2	B	15	5.3	no	6.4	a	1.0
	B	25	5.3	no	13.6	b	0.4
	B	40	5.3	no	14.5	b	0.7
3	B	25	5.3	no	13.9	a	1.1
	B	25	5.3	yes	8.9	b	1.1
	C	25	5.3	no	16.2	a	0.6
	C	25	5.3	yes	7.3	b	1.1

**Table 4:** Description of the nitrogen (potassium nitrate, ammonium chloride) and carbon (D-(+) glucose) sources supplied to the *Aspergillus niger* fungus in the different diets A, B, C and D.

	Carbon Sources	Nitrogen Sources
Diet A	20 g/L of D-(+) Glucose 1 (*δ*^13^C = −10.9‰)	2.5 g/L of potassium nitrate (*δ*^15^N = 1.3‰)
Diet B	20 g/L of D-(+) Glucose 2 (*δ*^13^C = −23.7‰)	2.5 g/L of ammonium chloride (*δ*^15^N = −1.8‰)
Diet C	20 g/L of D-(+) Glucose 1 (*δ*^13^C = −10.9‰)	2.5 g/L of ammonium chloride (*δ*^15^N = −1.8‰)
Diet D	20 g/L of D-(+) Glucose 2 (*δ*^13^C = −23.7‰)	2.5 g/L of potassium nitrate (*δ*^15^N = 1.3‰)

## Data Availability

Data will be made available on request from the corresponding author.

## Supplementary Materials

The following supporting information can be downloaded at: https://www.mdpi.com/article/10.3390/molecules30204142/s1, Table S1.
