# Supplementary material for: Influence of Diet and Growth Conditions on the Carbon and Nitrogen Stable Isotopic Composition of Aspergillus niger Mycelium: Insights for Fungal Chitosan Characterization
Source: Molecules. 2025 Oct 21;30(20):4142. doi: 10.3390/molecules30204142 (PMC12566308; doi:10.3390/molecules30204142)
Supplement: Supplementary file 1 [file molecules-30-04142-s001.zip › molecules-3884781-supplementary.pdf]

Table S1: Sample number (1-32), diet given to the *Aspergillus niger* fungus (Diet A-D), inoculum and sampling date, carbon (δ^{13}C‰, vs V-PDB) and nitrogen (δ^{15}N‰, vs AIR) isotopic ratios for the mycelium samples produced in this study; for each diet, the mean δ^{13}C and δ^{15}N are reported together with the standard deviation; different letters indicate statistically different results ($p<0.05$). All data were collected in 2023.

Sample	Diet	Date Inoculum	Date sampling	δ^{13}C (‰, vs V-PDB)		δ^{15}N (‰, vs AIR)	
1	A	22/4	2/5	-17.8		-4.4	
2	A	22/4	2/5	-18.2		-6.1	
3	A	22/4	2/5	-17.8		-5.6	
4	A	22/4	2/5	-17.5		-5.9	
5	A	22/4	2/5	-17.6		-5.5	
6	A	19/5	29/5	-17.6		-5.3	
7	A	19/5	29/5	-17.8		-5.6	
8	A	19/5	29/5	-17.6		-5.9	
9	A	19/5	29/5	-17.7		-6.3	
10	A	19/5	29/5	-17.7		-6.1	
			Mean	**-17.7**	**a**	**-5.7**	**a**
			St. Dev.	**0.2**		**0.5**	
11	B	22/4	2/5	-23.7		9.3	
12	B	22/4	2/5	-24.5		9.5	
13	B	22/4	2/5	-23.9		8.8	
14	B	22/4	2/5	-24.0		8.2	
15	B	22/4	2/5	-24.1		9.6	
16	B	19/5	29/5	-23.8		14.4	
17	B	19/5	29/5	-24.0		11.9	

18	B	19/5	29/5	-24.3		11.2	
19	B	19/5	29/5	-24.2		11.4	
20	B	19/5	29/5	-24.1		12.6	
			Mean	**-24.1**	**b**	**10.7**	**b**
			St. Dev.	**0.2**		**1.9**	
21	C	3/6	13/6	-18.2		10.0	
22	C	3/6	13/6	-18.3		9.5	
23	C	3/6	13/6	-18.2		9.2	
24	C	8/11	18/11	-17.7		10.0	
25	C	8/11	18/11	-17.6		9.8	
26	C	8/11	18/11	-17.7		10.6	
			Mean	**-18.0**	**a**	**9.9**	**b**
			St. Dev.	**0.3**		**0.5**	
27	D	3/6	13/6	-24.2		-1.3	
28	D	3/6	13/6	-24.2		-1.5	
29	D	3/6	13/6	-24.5		-2.5	
30	D	8/11	18/11	-24.2		-3.0	
31	D	8/11	18/11	-24.3		-2.5	
32	D	8/11	18/11	-24.3		-2.7	
			Mean	**-24.3**	**b**	**-2.3**	**c**
			St. Dev.	**0.1**		**0.7**	
